# Supplementary figures and images for: Phase diagrams of bone remodeling using a 3D stochastic cellular automaton
Source: PLoS One. 2024 Jun 11;19(6):e0304694. doi: 10.1371/journal.pone.0304694 (PMC11166309; doi:10.1371/journal.pone.0304694)

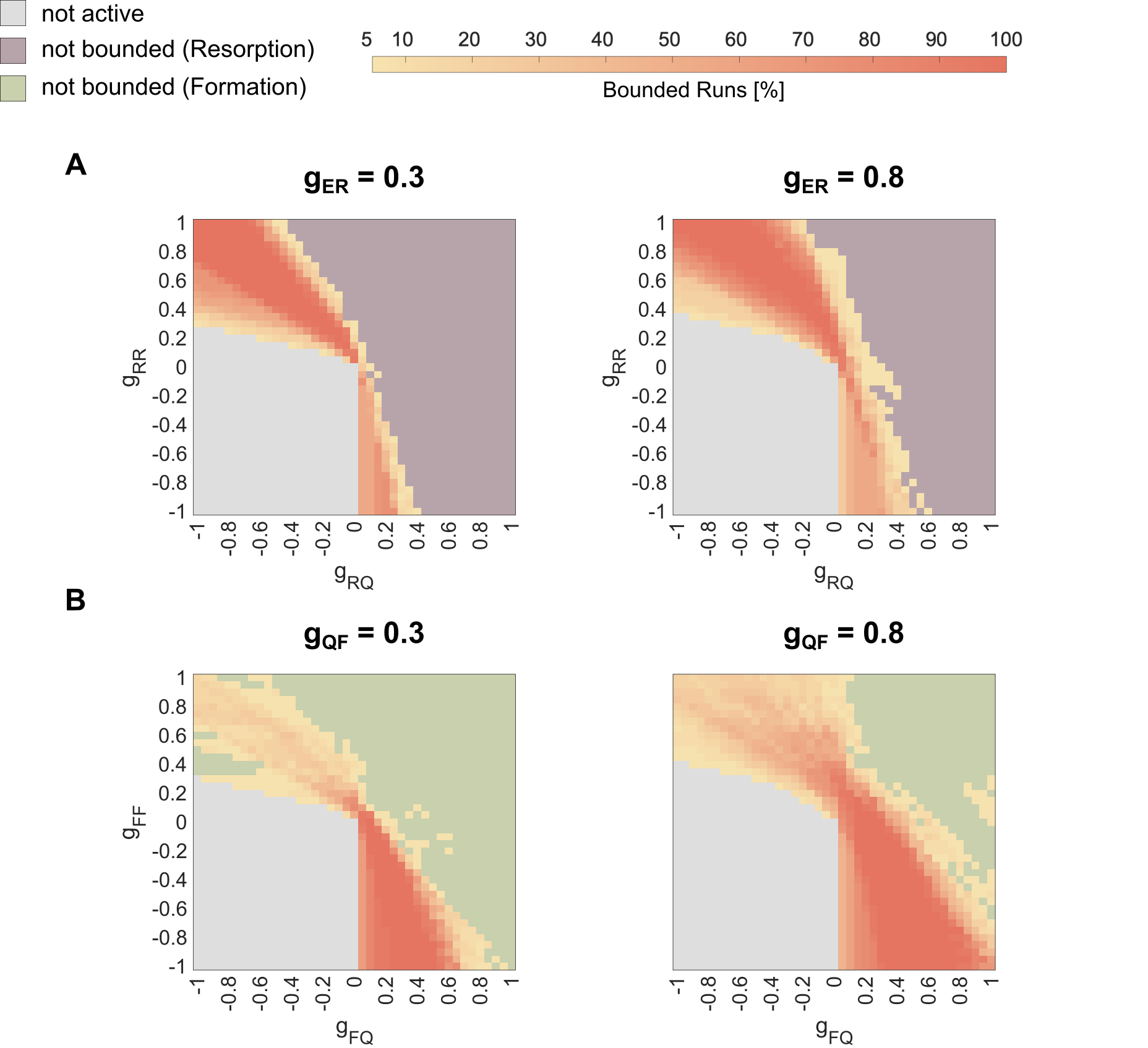

Supplement: S1 Fig — A: Bounded spreading phase for Resorption with gER = 0.3 (left) and gER = 0.8 (right) B: Bounded spreading phase for Formation with gQF = 0.3 (left) and gQF = 0.8 (right). (TIFF) [file pone.0304694.s001.tiff]

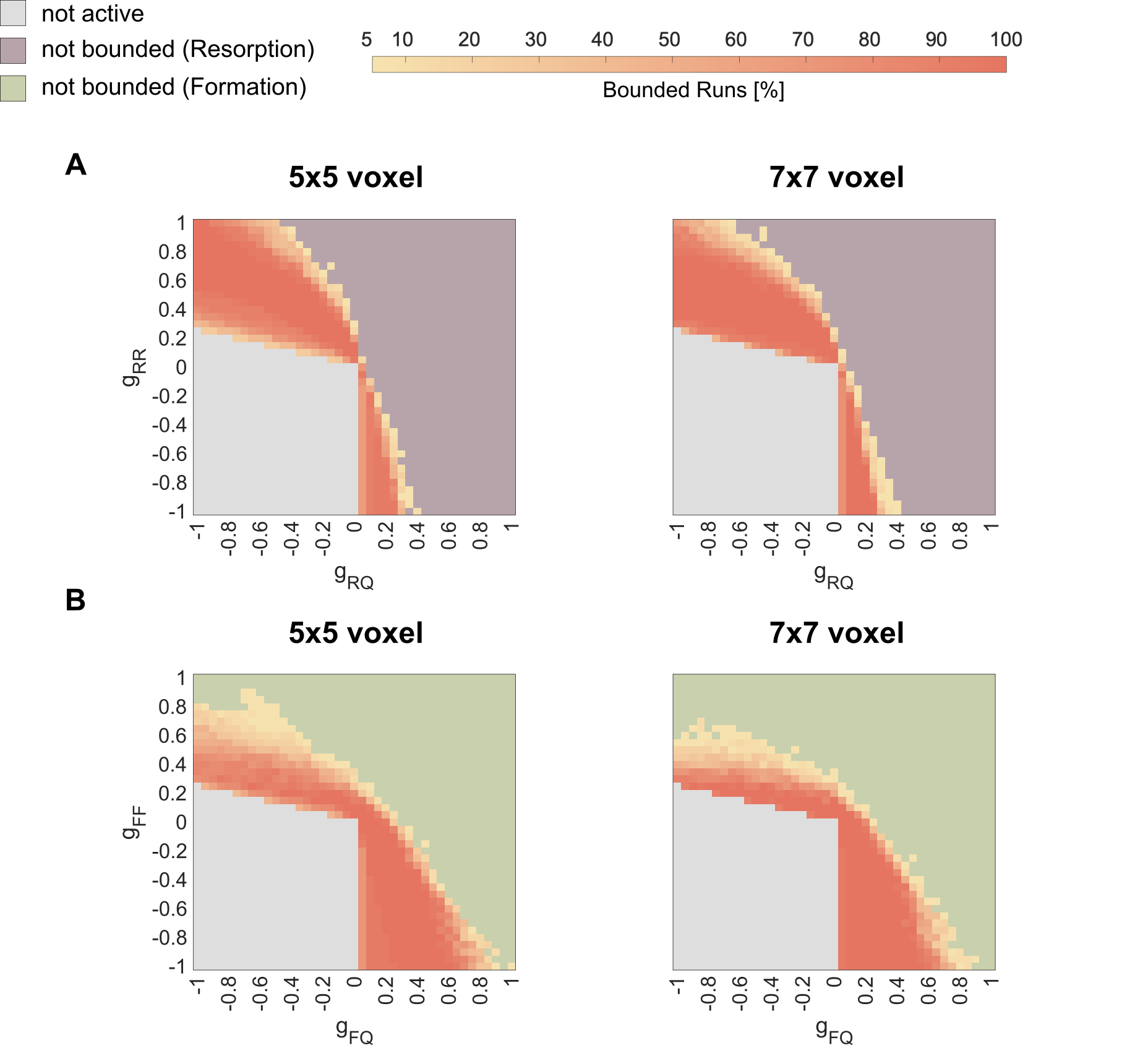

Supplement: S2 Fig — A: Bounded spreading phase for Resorption with a 5x5 voxel patch (left) and 7x7 voxel patch (right) B: Bounded spreading phase for Formation with a 5x5 voxel patch (left) and 7x7 voxel patch (right). (TIFF) [file pone.0304694.s002.tiff]

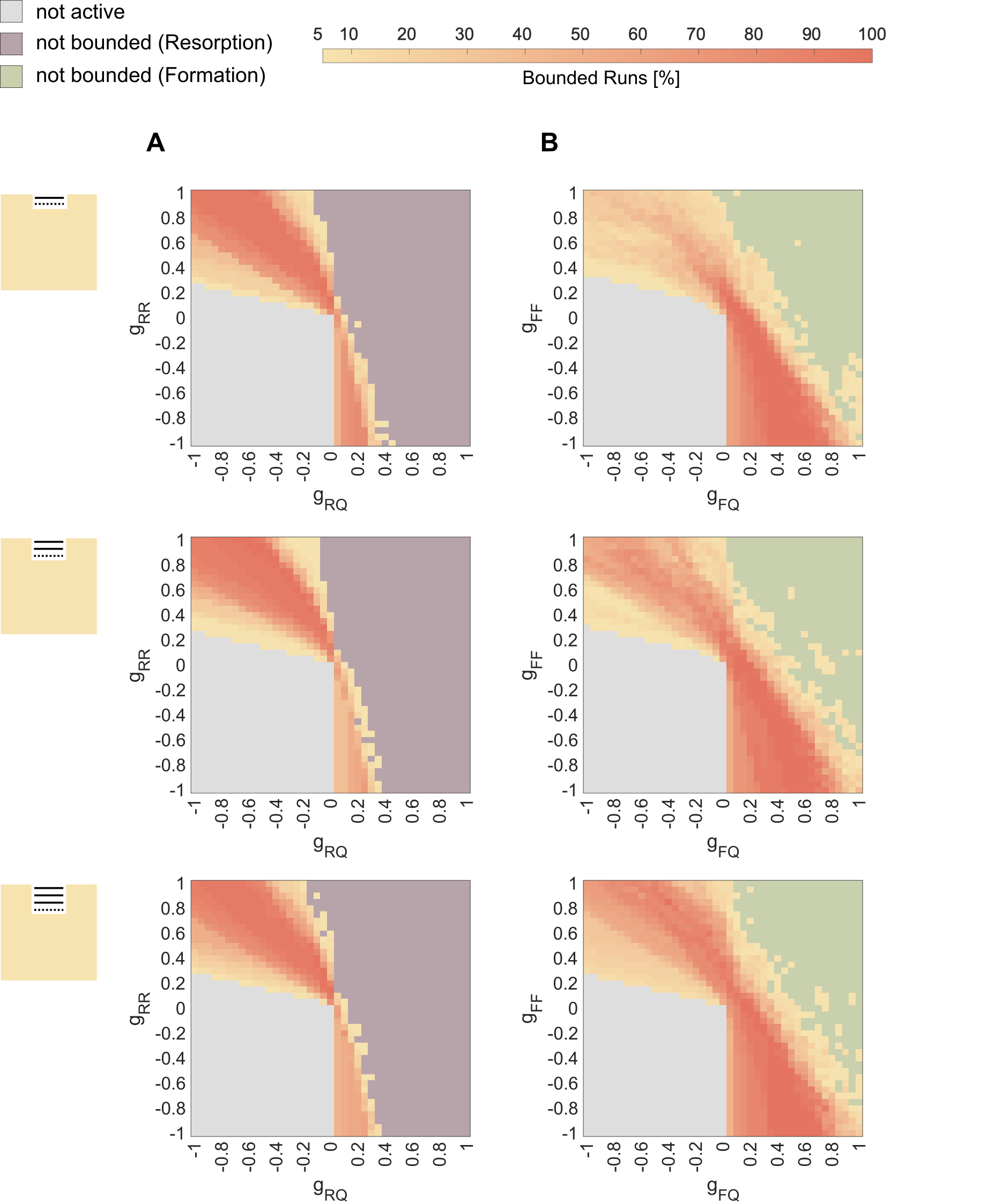

Supplement: S3 Fig — A: Bounded spreading phase for Resorption with the patch positioned one, two and three layers below surface (from top to bottom) B: Bounded spreading phase for Formation with the patch positioned one, two and three layers below surface (from top to bottom). (TIFF) [file pone.0304694.s003.tiff]

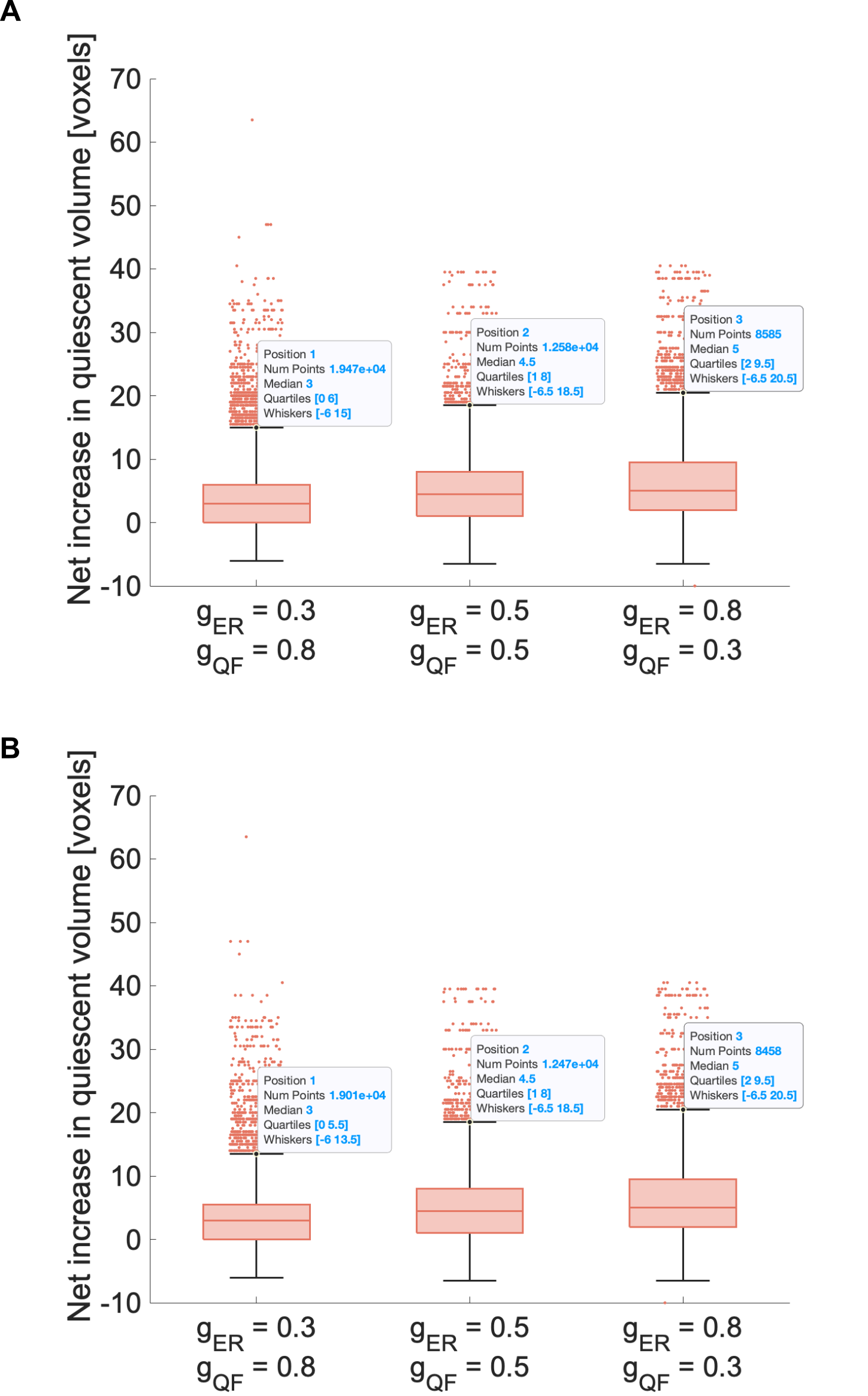

Supplement: S4 Fig — A: Distribution of net increase of volume for all parameter combinations (without exclusion) B: Distribution of net increase of volume excluding parameter combinations of unbounded runs. (TIFF) [file pone.0304694.s004.tiff]
